# Pilot study for a trial of ursodeoxycholic acid and/or early delivery for obstetric cholestasis

**DOI:** 10.1186/1471-2393-9-19

**Published:** 2009-05-16

**Authors:** Vinita Gurung, Catherine Williamson, Lucy Chappell, Jenny Chambers, Annette Briley, Fiona Broughton Pipkin, Jim Thornton

**Affiliations:** 1 School of Clinical Sciences, Division of Human Development, University of Nottingham, Nottingham, UK; 2 Institute of Reproductive and Developmental Biology, Imperial College London, London, UK; 3 Division of Reproduction and Endocrinology, King's College, London, UK

## Abstract

**Background:**

Obstetric cholestasis (OC) is a serious problem in pregnancy. It affects about 4500 women per year in the UK. Affected women develop itching and occasionally jaundice. More importantly, the condition is associated with premature delivery, fetal distress and is believed to be an important cause of stillbirth. However, even now, there is no clear evidence as to whether the most popular treatment, a drug called ursodeoxycholic acid is beneficial to the baby, or even if it is safe in pregnancy. Nor do we know whether planned early delivery of the baby at 37–38 weeks, another popular treatment, does more good than harm. A randomised trial to evaluate both ursodeoxycholic acid and timed delivery is needed but will be complicated and expensive. We plan a preliminary study, Pilot study for a trial of ursodeoxycholic acid and/or early delivery for obstetric cholestasis (Acronym PITCH- **P**regnancy **I**ntervention **T**rial in **Ch**olestasis) trial, to evaluate the feasibility of a larger trial. The trial is funded by the NHS Research for Patient Benefit (RfPB) Programme.

**Methods:**

PITCH is a multi-centre, double blinded, randomised, controlled, factorial design trial. The trial is being run in six UK centres and women with obstetric cholestasis will be recruited for eighteen months. In this pilot trial we aim to collect data to finalise the design for the main trial. This will include measuring trial recruitment rate, including recruitment to each factorial comparison separately. We will also measure the spectrum of disease among recruits and non-recruits and compliance with the four possible treatment allocations. We will use these data to design the main trial.

**Discussion:**

The ultimate aim of the main trial is to enable clinicians to manage this condition more effectively. If it transpires that ursodeoxycholic acid and early delivery are both safe and effective then steps will be taken to ensure that all women with OC who could benefit from them receives this treatment. Conversely, if one or both the treatments turn out to be ineffective or even harmful, they will be stopped and researchers will work at developing other modes of treatment.

**Trial registration number:**

ISRCTN37730443

## Background

Obstetric cholestasis (OC) is a liver condition unique to pregnancy. It is also referred to as Intrahepatic Cholestasis of Pregnancy (ICP). Pruritus is the characteristic symptom [1,2] accompanied by otherwise unexplained deranged liver enzymes [3-7] and elevated serum bile acid levels [3,7-9]. The itching subsides almost immediately after delivery [1,2,10] and the serum bile acid levels and the liver enzymes normalise within 2–3 weeks of delivery [1,10-14]. Occasionally, a patient might present with atypical symptoms such as jaundice, steatorrhoea, nausea, vomiting, anorexia, hepatomegaly and megalosplenia [15]. It is typically seen in the late second and third trimester [11,16,17] although some studies have reported presentation as early as six to ten weeks gestation [13,18].

Obstetric cholestasis affects between 0.7% and 1% [19]of pregnancies in the UK. It runs a relatively benign course in the mother. The main concern is its association with increased rates of perinatal mortality [1,5,11-14,17,20-24] and spontaneous preterm labour [11-13,17,20,21,23-25]

Most UK obstetricians treat obstetric cholestasis with ursodeoxycholic acid [26] in the belief that it reduces itching although this has not been shown in a suitably sized randomised controlled trial (RCT). Nor has fetal benefit or safety been adequately tested [27,28]. The latest Cochrane review, updated in 2001 [29] included three trials [30-32] involving 56 women comparing ursodeoxycholic acid with placebo. No statistically significant benefit on pruritus, or fetal outcomes was demonstrated. Since then a further trial [28] randomised 130 women diagnosed with obstetric cholestasis to placebo, ursodeoxycholic acid or dexamethasone. The only statistically significant effects in the groups as randomised were beneficial biochemical changes in the ursodeoxycholic acid group. In the subgroup with the highest bile acids at entry, ursodeoxycholic acid was associated with reduced pruritus. Although the only fetal death occurred in the placebo group, there was a non-significant trend towards more meconium in the ursodeoxycholic acid group. The latest guidelines from the RCOG (RCOG Guideline No. 43, January 2006) conclude as follows

"There are insufficient data to support the widespread use of ursodeoxycholic acid outside of clinical trials. Women should be aware of the lack of robust data concerning improvement in pruritus, protection against stillbirth and safety to the fetus or neonate."

Despite this lack of evidence about 4,500 pregnant women are treated with ursodeoxycholic acid each year in the UK and many more worldwide.

Delivery around 37–38 weeks is also widely practiced in the management of obstetric cholestasis on the assumption that it might pre-empt stillbirths [23]. Timed delivery is probably the single most important treatment offered by obstetricians. It is offered whenever the obstetrician believes that the risks of early delivery are less than those of awaiting labour. It is used in fetal growth restriction, pre-eclampsia, preterm and term rupture of membranes, diabetes, post maturity, placenta praevia and placental abruption. It has been evaluated in randomised controlled trials for post maturity, term and preterm rupture of membranes, pre-eclampsia and growth restriction. 75% of respondents in our survey indicated that they would deliver at least some patients with obstetric cholestasis early in the absence of fetal compromise. However, there have been no trials of such intervention and this practice has never been evaluated. There must be the potential for considerable risk of harm, including the fetal death or brain damage from iatrogenic prematurity, or of labour that is more prolonged than it otherwise would have been, and the maternal risks of increased operative delivery.

The RCOG guidelines (RCOG Guideline No. 43, January 2006) conclude as follows: *"Obstetricians should be aware that there are insufficient data to support or refute the popular practice of 'early' (37 weeks of gestation) induction of labour aimed at reducing late stillbirth."*

This is why both interventions need to be evaluated in an adequately powered randomised controlled trial. We are in the process of designing such a trial. Although we have had many indications of willingness to participate in such a trial, we are unable to finalise the sample size calculations without reliable data of the spectrum of disease severity which clinicians are prepared to recruit. To finalise the costings we also need a realistic recruitment rate per centre. We therefore plan "Pilot study for trial of ursodeoxycholic acid and/or early delivery for obstetric cholestasis"

## Methods and Study Design

In this pilot trial we aim to collect data to finalise the design of a factorial trial for the main trial. Ethical approval to conduct this trial was given by the Berkshire Research Ethics Committee (Reference number 08/H0505/7).

### Primary objective

• Measure recruitment to the two factorial interventions separately

### Secondary objectives

• Relate recruitment rates to disease severity

• Estimate a realistic recruitment rate for the definitive trial

• Measure acceptability of randomisation among potential participants offered trial entry

• Measure compliance with each treatment arm

• Measure the completeness of outcome data

• Finalise the design including the sample size calculation for the definitive trial

• Measure medium term (six weeks) maternal and fetal outcomes for the definitive trial.

PITCH is a multi-centre, double-blinded, randomised, controlled, factorial design trial. We will recruit in the three collaborating centres and three other UK centres for eighteen months. Centres will be asked to recruit participants with mild, moderate and severe obstetric cholestasis defined as follows:

Mild: random bile acids ≤ 14 μmol/L, ALT > 100 U/L

Moderate: random bile acids 15–40 μmol/L

Severe: random bile acids > 40 μmol/L

There are two comparisons:

### Comparison A – Ursodeoxycholic acid versus placebo

Investigational Medicinal Product (IMP) will be started at 500 mg bd. The dose will be increased in increments of 500 mg per day every 3–14 days if there is no biochemical or clinical improvement until a maximum of 2 grams per day is reached. Criteria for increasing the dose will be no improvement in itching or a rise in serum Alanine Transaminase (ALT) or bile acids. If there is no response to this dose, the dose can be increased up to 3 grams per day at the discretion of the treating clinician. The decision to increase the dose will always be at the discretion of the treating clinician.

#### Control group

Placebo capsule will be increased according to the same regime. In both control and intervention groups, participants will be blind to their group allocation.

Ursodeoxycholic capsules 250 mg and matching placebo are manufactured and supplied by Dr Falk Pharma GmbH. Packaging and labelling to provide blinded treatment packs is carried out in the production unit of the pharmacy department,, Queen's Medical Centre (QMC), Nottingham University Hospitals NHS Trust, UK. Supplies will be packed in an approved container and labelled with a single panel label. Subject name, randomisation number and date of dispensing will be added to the label as part of the dispensing process. The co-ordinating pharmacy will add a further label to each container to identify principal investigator, site address and contact telephone number.

### Comparison B – Early versus late delivery

Early planned delivery at between 37^+0 ^and 37^+6 ^weeks gestation by final agreed estimated date of delivery (EDD) or await spontaneous labour. For this part of the study, only participants <38^+0 ^weeks will be eligible. In early delivery/await spontaneous labour group, participants will be aware of group allocation.

We recognise that there is now evidence to support the induction of even uncomplicated pregnancy by 40^+10 ^weeks. Obstetricians are permitted to induce participants in the "await spontaneous labour" group from 40^+0 ^weeks, or as clinical needs dictate.

### Recruitment

Six UK centres will participate in the trial. Any pregnant women with itching and either a random serum bile acid level over 14 μmol/L, or an ALT over 100 U/L, between 24^+0 ^and 40^+6 ^weeks who has been diagnosed with obstetric cholestasis will be approached by a member of the clinical team and invited to participate in the trial. After seeking verbal consent, the potential participant will be referred to the local investigator. This local investigator may be a consultant, obstetrician in training or midwife according to local preferences. This person will give detailed information (verbal and the appropriate participant information sheet) regarding the purpose of the trial and procedures involved. If needed, the usual hospital interpreter and translator services will be available to assist with discussion of the trial, the participant information sheets, and consent forms but the consent forms and participant information sheets will not be available printed in other languages. Potential participants will be given adequate time to consider participation. Participants recruited at or after 38^+0 ^will only be eligible for ursodeoxycholic acid/placebo comparison. It will be explained to the potential participant that that entry into the trial is entirely voluntary and that their treatment and care will not be affected by their decision.

We will record all use of ursodeoxycholic acid, other drugs and labour induction for obstetric cholestasis outside the trial in participating centres.

### Inclusion criteria

• Itching in pregnancy

• 24^+0 ^to 37^+6 ^weeks pregnant eligible for both comparisons (38^+0 ^to 40^+6 ^weeks eligible for ursodeoxycholic acid/placebo comparison only)

• Bile acids > 14 μmol/L and/or ALT > 100 U/L

• Age 18 – 55 years

• Clinician responsible for care uncertain whether ursodeoxycholic acid or early delivery is beneficial

• Patients who otherwise fulfil the recruitment criteria, but incidentally have either hepatitis C, or cholelithiasis, or both, are eligible and may be included

• Women with multiple pregnancies who are otherwise eligible may be included in the ursodeoxycholic acid/placebo comparison only

• Willing to participate in the trial and able to give informed consent

### Exclusion criteria

• Dermatological and allergic pruritus with normal liver enzymes

• Other causes of pruritus and deranged liver enzymes (except hepatitis C and cholelithiasis)

• Hepatitis A, hepatitis B, pre-eclampsia, primary hepatic disorders, alpha-1 antitrypsin deficiency and current medications causing deranged liver enzymes

• Women unable or unwilling to consent

• Known lethal fetal anomalies

• Allergy to any component of the ursodeoxycholic acid or placebo capsules

### Consent

All participants will provide written informed consent. The consent form will be signed and dated by the participant before they enter the trial. The investigator will explain the details of the trial and provide the appropriate participant information sheet, ensuring that the participant has sufficient time to consider participation. The investigator will answer any questions that the participant has concerning study participation. One copy of the consent form will be kept by the participant, one will be kept by the investigator, and a third will be retained in the participant's hospital records. Should there be any subsequent amendment to the final protocol, which might affect a participant's participation in the trial, continuing consent will be obtained using an amended consent form which will be signed by the participant. The process for obtaining participant informed consent will be in accordance with the Research Ethics Committee (REC) guidance, and Good Clinical Practice (GCP) and any other regulatory requirements that might be introduced.

### Randomised procedure

After taking an informed consent, pre-randomisation baseline data will be collected (see additional file 1). The recruiting investigator (researcher, clinician or midwife) will ensure that a blood sample has been taken for a bile acid and ALT level. If convenient a fasting sample will be taken for bile acid. Participants will also be asked to consent for placenta and/or cord blood sample to be collected at delivery for DNA and molecular studies.

Randomisation will be via the Nottingham Clinical Trials Unit (NCTU) using a web-based database and randomisation system. In each centre, the recruiting investigator will have a username and password. S(he) will log on to the trial website that hosts the trial database, confirm that the participant eligibility criteria are all met and enter an agreed minimum amount of registration data about the participant and centre before randomisation is possible. The computer will then issue a participant study identification number which will be the unique identifier for the trial participant. Randomisation will be stratified by the trial centre only.

Participants will be randomised and allocated to ursodeoxycholic acid/placebo and/or early planned delivery/await spontaneous delivery (if < 38^+0 ^weeks). The details of the comparisons offered at each gestational age will differ as follows:

• 24^+0 ^– 34^+0 ^weeks:

• Offer ursodeoxycholic acid versus placebo immediately

• If still undelivered at 34^+1 ^– 37^+6 ^weeks, offer 'early delivery' versus 'await spontaneous labour'

• 34^+1 ^– 37^+6 ^weeks

Offer full factorial trial. Participants may choose one or the other comparison or both.

• Ursodeoxycholic acid versus placebo

• Early delivery versus await spontaneous labour

• ≥ 38^+0 ^weeks

• Offer ursodeoxycholic acid or placebo only

After randomisation to ursodeoxycholic acid/placebo comparison, an online prescription form will be generated. The investigator will download and print this prescription form. The participant is asked to collect the trial IMP/placebo from the pharmacy. The local pharmacist will select the pack with the appropriate number and issue this to the participant. The investigator, pharmacist and the trial participant will be blind to group allocation.

In early delivery/await spontaneous labour group, the investigator and the trial participant will be aware of group allocation. Obstetricians are permitted to induce participants in the 'await spontaneous labour' group from 40^+0 ^weeks, or as clinical needs dictate.

### Weekly follow-up

After randomisation, participants will be seen weekly by the researcher, a research midwife or their clinician as appropriate to local conditions in the antenatal clinic or day assessment unit. The participant will be asked to complete the compliance progress data form (see additional file 2) and the visual analogue itching scale. Blood samples will be collected weekly for ALT and bile acids and research blood samples will be collected for women who have consented. The frequency of fetal monitoring will depend on the local preferences. Each centre will define its monitoring regime.

The following data will be collected at weekly visit:

• Compliance progress data form

• Visual analogue itching scale

• Blood sample for serum bile acid and ALT level

• Use of other medications being taken by the participant

• Any adverse events

### Follow-up at or immediately after delivery

If the participant has consented to the optional genetic study, research samples (cord blood and placental sample) will be collected by the researcher, clinician or midwife as locally appropriate at the time of delivery. After delivery 'Outcome at hospital discharge' form (see additional file 3) will be completed by the local investigator.

### Follow-up at six weeks post delivery

**Figure 1:**
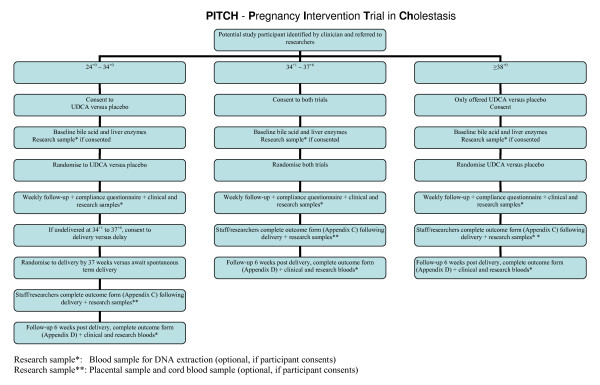
**Flow of trial participants from recruitment to six weeks follow-up after delivery**.

Study participants will be seen at six weeks post delivery in the antenatal clinic or day assessment unit by the investigator. The 'Outcome at six weeks post delivery' form (see additional file 4) will be completed during this visit. The visual analogue itching scale will be scored and a blood sample will be collected for serum bile acid and ALT level. Research samples will be collected if consented (figure 1).

### Withdrawal from the trial

Participants may withdraw from the trial either at their own request or at the discretion of the treating clinician. They will be made aware that this will not affect their future care. Participants will be made aware via the participant information sheet that should they withdraw, the data collected to date cannot be erased and may still be used in the final analysis. Participants who withdraw from treatment (either ursodeoxycholic acid or placebo; early delivery or await spontaneous labour) will remain in the studies for follow-up and analysis. Even those who decline to complete special follow-up arrangements will be asked to allow pregnancy outcome data to be included in the analysis and consent will be taken for the same. Those participants who wish to withdraw and are not yet randomised can be replaced but participants who withdraw after randomisation will not be replaced.

### Maintenance of randomisation codes and procedures for breaking code

The placebos used will be similar in appearance to the IMP capsules. They will be provided in bottles. There will be no marking on the capsule to suggest whether it is a placebo or IMP. If the participants clinical condition deteriorates (worsening of itching, increasing bile acids and/or ALT), the patients clinician can make the decision for delivery as clinical needs dictate. The treatment code will not be broken in such cases. In cases of serious adverse event (SAE), the event shall be reported immediately of knowledge of its occurrence to the chief investigator.

### Endpoints

#### Primary endpoint

• Total recruitment rate per 1000 deliveries/annum

• Total recruitment rate per eligible women

#### Secondary endpoint

• Recruitment for the three subgroups of mild, moderate and severe disease separately

#### Safety endpoints

It is not possible to define a predetermined safety endpoint for this trial. Both treatments are in common use. All adverse events will be reported to the Data Monitoring and Ethics Committee (DMEC).

### Statistics

We have estimated that a provisional sample size for the main factorial trial will be 1498 women (749 per group). This would give 80% power, alpha 0.05, to show a reduction in the primary composite endpoint (fetal death or severe morbidity) from 6% to 3%. However, detailed power calculations are not possible without measuring the clinical spectrum of likely recruits to such a trial.

In the pilot phase we will develop and pilot the definitive trial as follows:

At the launch meeting, collaborators will register their current obstetric cholestasis treatment policies. All collaborators will also agree to register all cases of obstetric cholestasis in their centre. Collaborators will then return to their centres and recruit to the trial for eighteen months. During this time each centre will receive three site visits from the research fellow, as well as regular newsletters, email and phone contacts. At the end of the pilot recruitment period each centre will produce the following data:

• Total number of deliveries

• Number of participants identified with obstetric cholestasis in each severity band

• Number recruited to the full factorial trial, and to the ursodeoxycholic acid/placebo or the early delivery/await spontaneous labour comparison only

• Number treated for obstetric cholestasis outside the trial

• Compliance with treatment and outcome data as for the definitive trial.

In the pilot phase we will recruit in each centre for eighteen months. We recognise that strictly the most efficient way to measure the recruitment rate in each centre would be to recruit until a fixed number of participants, say ten, had been enrolled. However, given the sunk costs of setting up each centre, there are little extra costs from continuing each centre beyond such a number. The target sample size of the pilot of approximately ninety participants (equivalent to fifteen particpants per centre over eighteen months, ten per centre per annum) has been chosen to allow a reasonably precise estimate of the various parameters for the main trial.

1. Mean recruitment rate per centre over eighteen months. By weighting recruitment by the size of each unit we will model predicted recuitment for the proposed centres in the main trial.

2. The proportion of moderate/severe cases. If this is much below 50% the main trial will probably not be feasible. Example 45/90 50% severe cases would have 95% CI of 39–61%.

3. The proportion recruiting to both comparison groups. Example 80/90 89% 95% CI 81–94%

We cannot at this stage specify exactly what levels for each parameter would make the main trial feasible since to some extent they can be traded off against each other. For example a relatively low level of severe cases recruited can be traded off against a higher than expected overall recruitment rate. The following broad parameters are suggested.

#### Example 1

Recruitment rate per centre of 10 per annum, of which 2/3 moderate or severe cases, >80% enter both comparison arms, >95% compliance with ursodeoxycholic acid/placebo comparison and >70% compliance with early delivery/await spontaneous labour comparison. We would plan to recruit for the main trial in 50 centres over three years. We believe that this would probably be feasible within the UK

#### Example 2

Recruitment rate per centre of 5 per annum. Same proportion of severe cases and same compliance levels would require a trial with 100 centres over three years. This would probably be too much for UK only centres. We would consider collaboration with another one or two European Union countries on a similar trial design.

#### Example 3

Recruitment rate per centre of less than 5 per annum. Only 1/3 moderate or severe. The main trial would not be realistically feasible. Similarly if recruitment to either the ursodeoxycholic/placebo comparison or to the earlydelivery/await spontaneous labour comparison was below 50% we would probably drop the relevant comparison from the main trial.

We have intentionally not drawn hard and fast rules for going ahead or not with the main trial. At the end of the pilot we will call a collaborators meeting of representatives of about 50 UK centres at the RCOG at which the pilot data will be presented, a range of trial designs will be presented, feasibility will be discussed and a final decision will be taken.

### Adverse events

An adverse event (AE) is any unfavourable and unintended sign, symptom, syndrome or illness that develops or worsens during the period of observation in the study. The following will be considered adverse events:

• Fetal death

• Maternal death

An adverse event will not include:

• Itching

• Admission to hospital for delivery or fetal monitoring or operative delivery

• Congenital anomaly in the offspring of a participant in the current pregnancy

• Admission to special care baby unit (SCBU)

• A disability or incapacity

A Serious Adverse Event (SAE) is any adverse event occurring following study mandated procedures, having received ursodeoxycholic acid or placebo or waited for spontaneous term delivery that results in any of the following outcomes:

• Death

• A life-threatening adverse event

• Inpatient hospitalisation or prolongation of existing hospitalisation: Admission for delivery or fetal monitoring, induction of labour or operative deliveries in which no fetal or maternal complication occurs will not be regarded as SAE.

• A disability/incapacity

• A congenital anomaly in the offspring of a participant in the present pregnancy

Important medical events that may not result in death, be life-threatening, or require hospitalisation may be considered a SAE when, based upon appropriate medical judgment, they may jeopardise the study participant and may require medical or surgical intervention to prevent one of the outcomes listed in this definition. All adverse events will be assessed for seriousness, expectedness and causality.

#### Seriousness

A distinction is drawn between serious and severe adverse events. Severity is a measure of intensity whereas seriousness is defined using the criteria above. Hence, a severe adverse event need not necessarily be serious.

#### Causality

*Unrelated or improbable*: a clinical event including laboratory test abnormality with temporal relationship to trial treatment administration which makes a causal relationship incompatible or for which other drugs, chemicals or disease provide a plausible explanation. This will be counted as 'unrelated' for notification purposes.

*Possible*: a clinical event, including laboratory test abnormality, with temporal relationship to trial treatment administration which makes a causal relationship a reasonable possibility, but which could also be explained by other drugs, chemicals or concurrent disease. This will be counted as 'related' for notification purposes.

*Probable*: a clinical event, including laboratory test abnormality, with temporal relationship to trial treatment administration which makes a causal relationship a reasonable possibility, and is unlikely to be due to other drugs, chemical or concurrent disease. This will be counted as 'related' for notification purposes.

*Definite*: a clinical event, including laboratory test abnormality, with temporal relationship to trial treatment administration which makes a causal relationship a reasonable possibility, and which can definitely not be attributed to other causes. This will be counted as 'related' for notification purposes.

An adverse event whose causal relationship to the study IMP is assessed by the chief investigator as 'possible', 'probable', or 'definite' is an Adverse Drug Reaction (ADR). Medical and scientific judgment shall be used in deciding whether prompt reporting is appropriate in that situation.

#### Reporting of adverse events

Participants will be asked to contact the study site immediately in the event of any serious adverse event. All adverse events will be recorded and closely monitored until resolution, stabilisation, or until it has been shown that the study medication or treatment is not the cause. The Chief Investigator shall be informed immediately of any serious adverse events and shall determine seriousness and causality in conjunction with any treating medical practitioners. NCTU standard operating procedure (SOP) for adverse event reporting will be followed.

All serious adverse events will be recorded and reported to the Medicines and Healthcare products Regulatory Agency (MHRA) and Research Ethics Committee (REC) as part of the annual reports. Suspected Unexpected Serious Adverse Reactions (SUSARs) will be reported within the statutory timeframes to the MHRA and REC, as stated below. The Chief Investigator shall be responsible for all adverse event reporting.

#### Suspected Unexpected Serious Adverse Reactions (SUSARs)

A serious adverse event that is sudden in its onset, unexpected in its severity and seriousness or not a known side effect of the IMP and related or suspected to be related to the IMP is classed as SUSAR and requires expedited reporting as per the clinical trials regulations. All serious adverse events that fall or are suspected to fall within these criteria shall be treated as a SUSAR until deemed otherwise. The event shall be reported immediately of knowledge of its occurrence to the Chief Investigator.

The Chief Investigator will:

• Assess the event for seriousness, expectedness and relatedness to the study IMP

• Take appropriate medical action, which may include halting the trial and inform the sponsor of such action

• If the event is deemed a SUSAR, shall, within seven days, complete the Council for International Organizations and Medical Sciences (CIOMS) form and send to the MHRA

• Shall inform the REC using the reporting form found on the National Research Ethics Service (NRES) web page within seven days of knowledge of the event

• Shall, within a further eight days send any follow-up information and reports to the MHRA and REC.

• Make any amendments as required to the study protocol and inform the ethics and regulatory authorities as required

#### Participant removal from the study due to adverse events

Any participant who experiences an adverse event may be withdrawn from the study at the discretion of the Chief Investigator.

### Ethics committee and regulatory approvals

The trial will not be initiated before the protocol, informed consent forms and participant information sheets and GP letter have received approval/favourable opinion from the MHRA, REC, and the respective National Health Service (NHS) Research & Development (R&D) department. Should a protocol amendment be made that requires REC approval, the changes in the protocol will not be instituted until the amendment and revised informed consent forms and participant information sheets and GP letter have been reviewed and received approval/favourable opinion from the REC and R&D departments. A protocol amendment intended to eliminate an apparent immediate hazard to participants may be implemented immediately providing that the MHRA, R&D and REC are notified as soon as possible and an approval is requested. Minor protocol amendments only for logistical or administrative changes may be implemented immediately; and the REC will be informed.

The trial will be conducted in accordance with the ethical principles that have their origin in the Declaration of Helsinki, 1996; the principles of Good Clinical Practice (GCP) and in accordance with the Medicines for Human Use Regulations, Statutory Instrument 2004, 1031 and its subsequent amendments.

### Records

#### Drug accountability

Drug supplies will be kept in a secure, limited access storage area under the storage conditions specified by the pharmacy. The investigator and the local site pharmacist shall maintain records of the study drug's delivery to the pharmacy, an inventory at the site, the distribution to each participant, and the return to the pharmacy or alternative disposition of unused study drugs. These records will include dates, quantities received, batch/serial numbers, expiration dates, and the participant study identification number assigned to the trial participant. Investigators and/or the local site pharmacists will maintain records that document adequately that the participants were provided with the correct study medication. These records will be part of each participant's Case Report Form (CRF). All study medication packs and bottles received by the pharmacy shall be accounted for.

#### Case Report Forms

Each participant will be assigned a participant study identity code number, allocated at randomisation, for use on CRFs, other trial documents and the electronic database. The documents and database will also use their initials (of first and last names separated by a hyphen or a middle name initial when available) and date of birth (dd/mm/yy). CRFs will be treated as confidential documents and held securely in accordance with regulations. The investigator will make a separate confidential record of the participant's name, date of birth, local hospital number or NHS number, and participant study identification number to permit identification of all participants enrolled in the trial, in case additional follow-up is required. CRFs shall be restricted to those personnel approved by the Chief or local Principal Investigator and recorded on the Trial Delegation Log (TDL). All paper forms shall be filled in using black ballpoint pen. Errors shall be lined out but not obliterated by using correction fluid and the correction inserted, initialled and dated. The Chief or local Principal Investigator shall sign a declaration ensuring accuracy of data recorded in the CRF.

#### Source documents

Source documents shall be filed at the investigator's site and may include but are not limited to, consent forms, current medical records, laboratory results and pharmacy records. A CRF may also completely serve as its own source data. Only trial staff as listed on the TDL shall have access to trial documentation other that the regulatory requirements listed below.

#### Direct access to source data/document

The CRF and all source documents, including progress notes and copies of laboratory and medical test results shall made be available at all times for review by the Chief Investigator, Sponsor's designee and inspection by relevant regulatory authorities.

### Data protection

All trial staff and investigators will endeavour to protect the rights of the trial participants to privacy and informed consent, and will adhere to the Data Protection Act, 1998. The CRF will only collect the minimum required information for the purposes of the trial. CRFs will be held securely, in a locked room, or locked cupboard or cabinet. Access to the information will be limited to the trial staff and investigators and relevant regulatory authorities. Computer held data including the trial database will be held securely and password protected. All data will be stored on a secure dedicated web server. Access will be restricted by user identifiers and passwords (encrypted using a one way encryption method). Information about the trial in the participant's medical records/hospital notes will be treated confidentially in the same way as all other confidential medical information. Electronic data will be backed up every 24 hours to both local and remote media in encrypted format.

#### Trial management

The management of the PITCH trial includes an element of advice that is completely independent from the principal investigators and their host institutions. We shall appoint a Trial Steering Committee (TSC) including an independent chair, an independent expert member and a lay member. The TSC will provide overall supervision of the trial, in particular, trial progress, adherence to the protocol, patient safety and the consideration of new information.

A Data Monitoring and Ethics Committee (DMEC) will be established. Members of DMEC will meet regularly to view data and the results of any interim analyses. During the pilot phase we propose that there be no formal stopping rules. However, the DMEC will review unblinded data at least annually together with all reported adverse drug reactions as they accrue. We will recruit to the pilot study for eighteen months. It will cease earlier if the DMEC recommends stopping. DMEC members will be independent of both the trial and TSC.

### Insurance and indemnity

Insurance and indemnity for trial participants and trial staff is covered within the NHS Indemnity Arrangements for clinical negligence claims in the NHS, issued under cover of HSG (96)48. There are no special compensation arrangements, but trial participants may have recourse through the NHS complaints procedures.

### Trial conduct

Trial conduct will be subject to systems audit of the Trial Master File (TMF) for inclusion of essential documents; permissions to conduct the trial; TDL; curriculum vitae of trial staff and training received; local document control procedures; consent procedures and recruitment logs; adherence to procedures defined in the protocol (e.g. inclusion/exclusion criteria, correct randomisation, timeliness of visits); adverse event recording and reporting; drug accountability and pharmacy records. The Trial Manager, or where required, a nominated designee of the Sponsor, shall carry out a site systems audit at least yearly and an audit report shall be made to the TSC.

### Trial data

Monitoring of trial data shall include confirmation of informed consent; source data verification; data storage and data transfer procedures; local quality control checks and procedures, back-up and disaster recovery of any local databases and validation of data manipulation. The Trial Manager, or where required, a nominated designee of the Sponsor, shall carry out monitoring of trial data as an ongoing activity.

Entries on CRFs will be verified by inspection against the source data. A sample of CRFs (10%) will be checked on a regular basis for verification of all entries made. In addition the subsequent capture of the data on the trial database will be checked. Where corrections are required these will carry a full audit trail and justification. Trial data and evidence of monitoring and systems audits will be made available for inspection by the regulatory authority as required.

### Record retention and archiving

In compliance with the ICH/GCP guidelines, regulations and in accordance with the University of Nottingham Research Code of Conduct, the Chief or local Principal Investigator will maintain all records and documents regarding the conduct of the study. These will be retained for at least seven years or for longer if required. If the responsible investigator is no longer able to maintain the study records, a second person will be nominated to take over this responsibility.

The TMF and trial documents held by the Chief Investigator on behalf of the Sponsor shall be finally archived at secure archive facilities at the University of Nottingham. This archive shall include all trial databases and associated meta-data encryption codes.

### Discontinuation of the trial by the sponsor

The Sponsor reserves the right to discontinue this trial at any time for failure to meet expected enrolment goals, for safety or any other administrative reasons. The sponsor shall take advice from the TSC and DMEC as appropriate in making this decision.

### Statement of confidentiality

Individual participant medical information obtained as a result of this study is considered confidential and disclosure to third parties is prohibited with the exceptions noted above. Participant confidentiality will be further ensured by utilising participant study identification numbers to correspond to treatment data in the computer files.

Such medical information may be given to the participant's medical team and all appropriate medical personnel responsible for the participant's welfare. Data generated as a result of this trial will be available for inspection on request by the participating physicians, the University of Nottingham representatives, the REC, local R&D Departments and the regulatory authorities.

### Funding source

The NHS Research for Patient Benefit programme (East Midlands) and Nottingham University Hospitals NHS Trust are funding the pilot study. Funding is being administered by the University of Nottingham.

### Participant stipends and payments

Participants will not be paid to participate in the trial. Each centre will be paid £300 per participant for the expenses.

## Abbreviations

ADR: Adverse Drug Reaction; AE: Adverse Event; ALT: Alanine Transaminase; CI: Chief Investigator; CIOMS: Council for International Organizations and Medical Sciences; CRF: Case Report Form; CTA: Clinical Trial Authorisations; CTG: Cardiotocography; DGH: District General Hospital; DMEC: Data Monitoring and Ethics Committee; EDD: Estimated Date of Delivery; GCP: Good Clinical Practice; GGT: Gamma Glutamyl Transferase; GP: General Practitioner; ICF: Informed Consent Form; ICH: International Conference on Harmonization; IMP: Investigational Medicinal Product; MHRA: Medicines and Healthcare products Regulatory Agency; NCTU: Nottingham Clinical Trials Unit; NHS: National Health Service; NRES: National Research Ethics Service; OC: Obstetric Cholestasis; PI: Principal Investigator; PIS: Participant Information Sheet; PITCH: Pregnancy Intervention Trial in Cholestasis; RCOG: Royal College of Obstetricians and Gynaecologists; RCT: Randomised Controlled Trial; REC: Research Ethics Committee; R&D: Research and Development department; RFPB: Research for Patient Benefit; SAE: Serious Adverse Event; SAR: Serious Adverse Reaction; SCBU: Special Care Baby Unit; SOP: Standard Operating Procedure; SUSAR: Suspected Unexpected Serious Adverse Reaction; TDL: Trial Delegation Log; TMF: Trial Master File; TMG: Trial Management Group; TSC: Trial Steering Committee; UDCA: Ursodeoxycholic acid.

## Competing interests

The authors declare that they have no competing interests.

## Authors' contributions

All the authors have made substantial contributions to conception and design of the protocol. They have been involved in drafting the manuscript and revising it critically for important intellectual content. They have given final approval of the version to be published.

## Pre-publication history

The pre-publication history for this paper can be accessed here:



## Supplementary Material

Additional file 1 **Baseline data form**. The baseline data collected prior to randomisation. Click here for file

Additional file 2 **Compliance progress data form**. The compliance data collected weekly until delivery. Click here for file

Additional file 3 **Outcome at hospital discharge form**. The data collected after delivery at the time of hospital discharge. Click here for file

Additional file 4 **Outcome at six weeks post delivery form**. The data collected at six weeks post delivery follow-up visit. Click here for file
