# Supplementary figures and images for: Pilot study for a trial of ursodeoxycholic acid and/or early delivery for obstetric cholestasis
Source: BMC Pregnancy Childbirth. 2009 May 16;9:19. doi: 10.1186/1471-2393-9-19 (PMC2696408; doi:10.1186/1471-2393-9-19)

**Baseline data**


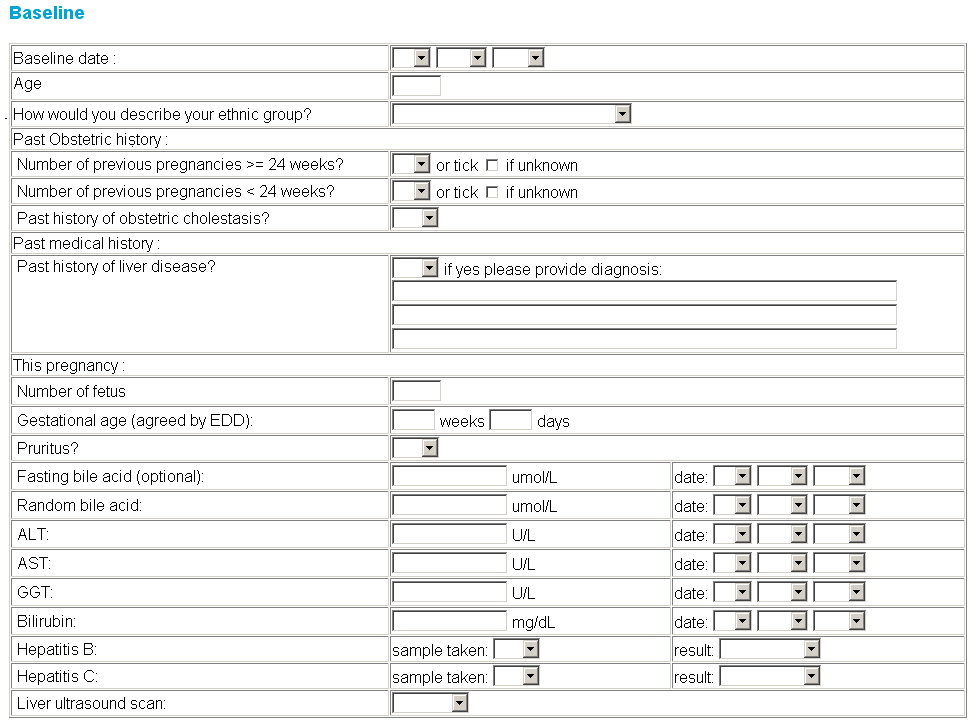

Supplement: Additional file 1 — Baseline data form. The baseline data collected prior to randomisation. [file 1471-2393-9-19-S1.docx]

**
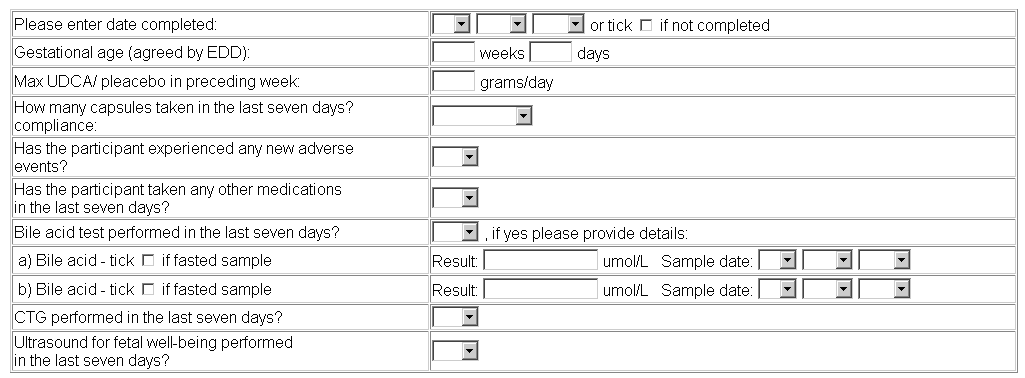
Compliance progress data form**

Supplement: Additional file 2 — Compliance progress data form. The compliance data collected weekly until delivery. [file 1471-2393-9-19-S2.docx]

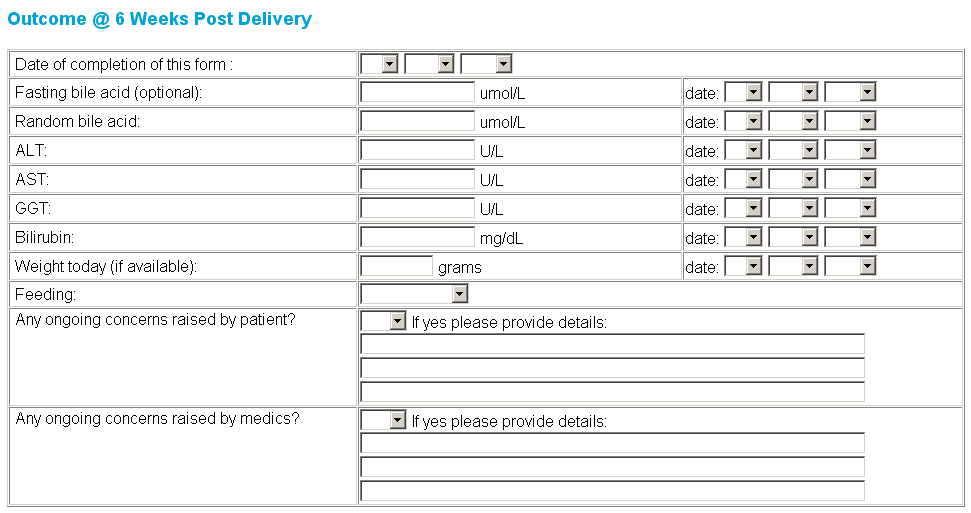


Feeding: breast/bottle/breast + bottle

Supplement: Additional file 4 — Outcome at six weeks post delivery form. The data collected at six weeks post delivery follow-up visit. [file 1471-2393-9-19-S4.docx]
