# Supplementary material for: Pilot study for a trial of ursodeoxycholic acid and/or early delivery for obstetric cholestasis
Source: BMC Pregnancy Childbirth. 2009 May 16;9:19. doi: 10.1186/1471-2393-9-19 (PMC2696408; doi:10.1186/1471-2393-9-19)
Supplement: Additional file 3 — Outcome at hospital discharge form. The data collected after delivery at the time of hospital discharge. [file 1471-2393-9-19-S3.docx]

*
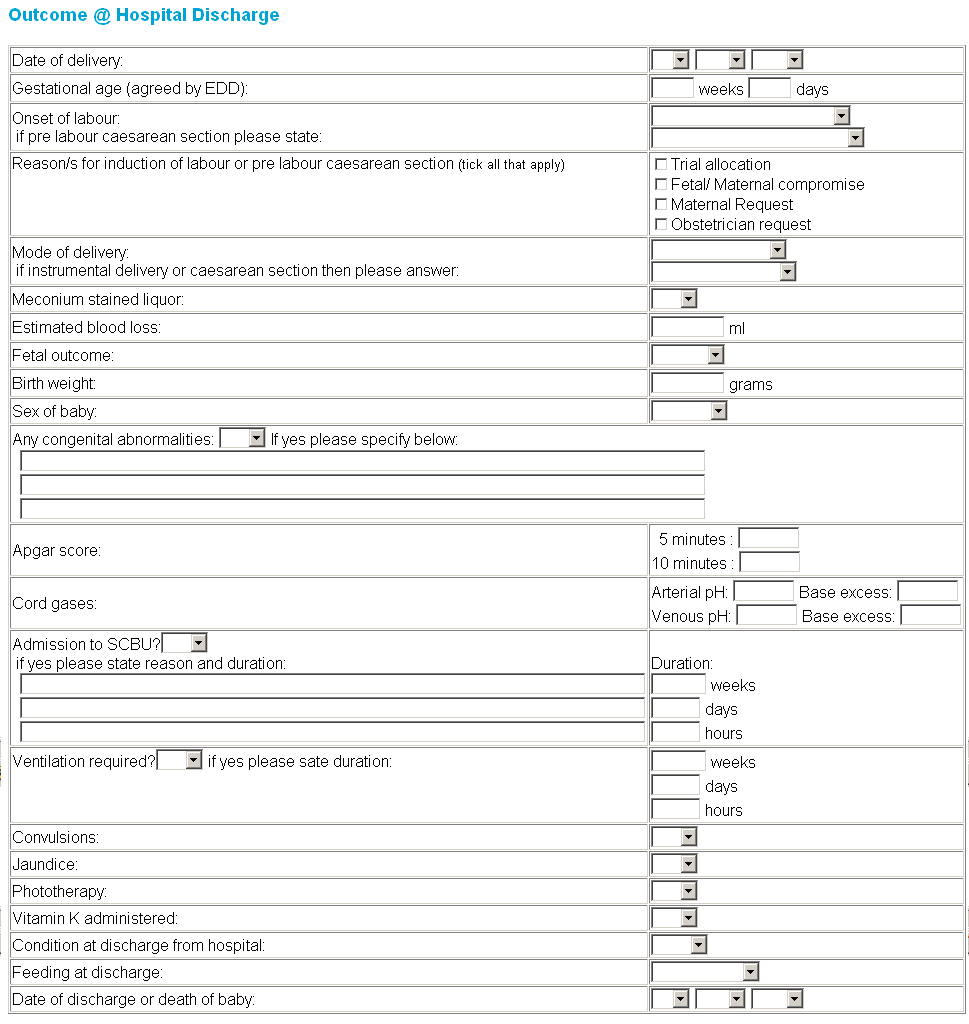
*

Feeding at discharge: breast/bottle/breast + bottle

Onset of labour: spontaneous/induced/pre labour caesarean section

If pre labour caesarean section: elective/ emergency

Mode of delivery: normal vaginal/ assisted vaginal/ caesarean section

If instrumental delivery or caesarean section: fetal compromise/ no fetal compromise

Condition of discharge from hospital: live/ dead
